# Birds of a Feather Flock Together: Experience-Driven Formation of Visual Object Categories in Human Ventral Temporal Cortex

**DOI:** 10.1371/journal.pone.0003995

**Published:** 2008-12-24

**Authors:** Marieke van der Linden, Jaap M. J. Murre, Miranda van Turennout

**Affiliations:** 1 F.C. Donders Centre for Cognitive Neuroimaging, Nijmegen, The Netherlands; 2 Department of Psychology, University of Amsterdam, Amsterdam, The Netherlands; 3 Behavioural Science Institute, University of Nijmegen, Nijmegen, The Netherlands; Centre de Recherches su la Cognition Animale - Centre National de la Recherche Scientifique and Université Paul Sabatier, France

## Abstract

The present functional magnetic resonance imaging study provides direct evidence on visual object-category formation in the human brain. Although brain imaging has demonstrated object-category specific representations in the occipitotemporal cortex, the crucial question of how the brain acquires this knowledge has remained unresolved. We designed a stimulus set consisting of six highly similar bird types that can hardly be distinguished without training. All bird types were morphed with one another to create different exemplars of each category. After visual training, fMRI showed that responses in the right fusiform gyrus were larger for bird types for which a discrete category-boundary was established as compared with not-trained bird types. Importantly, compared with not-trained bird types, right fusiform responses were smaller for visually similar birds to which subjects were exposed during training but for which no category-boundary was learned. These data provide evidence for experience-induced shaping of occipitotemporal responses that are involved in category learning in the human brain.

## Introduction

A crucial property of the human object-recognition system is its capacity to group different-looking objects into the same category, and to assign similar-looking objects to different categories. Pineapples and berries look very different, but they are both members of the category ‘fruits’. In contrast, berries and beads can look similar, but belong to different categories. Someone more skilled in recognizing fruits might be able to discriminate between similar sub-exemplars of berries (e.g., salmonberries and raspberries), suggesting that the neural representation of object categories is plastic and changes as a result of experience. The present study investigates the neural mechanisms mediating experience-induced formation of visual object categories in the human brain.

There are strong indications both from neuropsychological and functional brain imaging experiments that the ventral temporal cortex is involved in the representation of category-specific information [1], [2], [3], [4]. Differential neural responses within occipitotemporal cortex have been demonstrated for a wide range of object categories [5], [6], [7], [8]. However, the neural mechanisms mediating the formation of category-specific representations in human occipitotemporal cortex are still largely unknown. Animal studies have revealed that learning and experience can shape neural response properties of cells in inferior temporal cortex, possibly resulting in category-specific representations. For example, after monkeys were trained to categorize visual stimuli, inferior temporal neurons responded selectively to stimuli belonging to the trained category [9]. Furthermore, other electrophysiological recordings from monkey cortex revealed increased selectivity in responses from inferior temporal neurons for visual stimulus features diagnostic for trained object categories [10], as well as for combinations of features in learned objects [11]. Functional imaging of the human brain has shown that visual as well as functional experience with novel object categories alters neural responses in occipitotemporal cortex [12], [13], [14], [15]. Recently, fMRI data provided evidence for increased neural sensitivity in occipitotemporal cortex after categorization training [16]. It remains unclear, however, whether, and how training-related neuronal changes are linked to the formation of behaviourally relevant object categories.

In the present study, we investigate neural mechanisms of object category formation in human occipitotemporal cortex. We directly compare neural changes mediating the formation of behaviourally relevant object categories with neural changes following visual exposure to objects in the absence of category formation. Our findings provide evidence for learning-related increases in selectivity of neural responses to object properties that are relevant for categorization.

**Figure 1:**
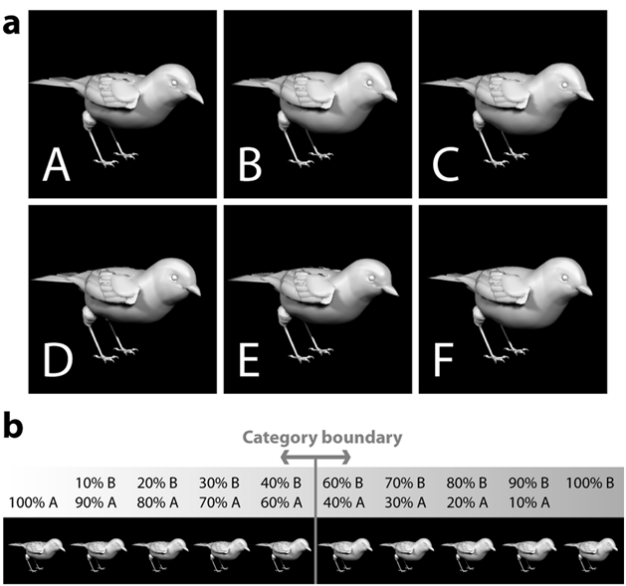
Construction of the stimulus set. (A) Pictures of non-existing but plausible bird shapes were constructed in a 3D model manipulation program. From a base-bird we derived six colorless prototype birds (A, B, C, D, E, F) that differed in trunk, belly, tail, beak, head shape, cheeks, brow, and eye position. Each bird was rendered under the same lighting and camera settings to make sure that shading and scale was identical for all birds. (B) Exemplars were created by systematically morphing each of the six prototype birds with all other birds. Shown is an example of morphing bird type A and bird type B at morph ratios of 90∶10, 80∶20, 70∶30, 60∶40. The category boundary was set at 50∶50.

**Figure 2:**
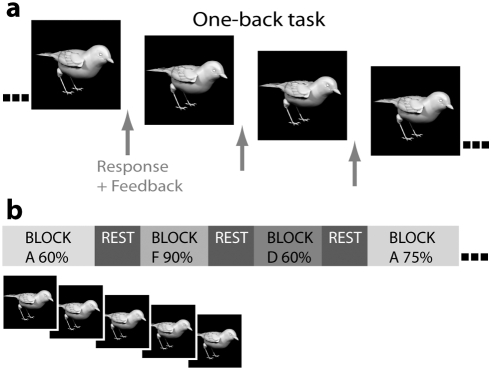
Training and fMRI paradigms. (A) During the training sessions participants were presented with a series of bird exemplars. They performed a 1-back task in which they indicated whether two consecutive birds were the same type or not. In the category-training condition implicit category learning was established by providing corrective feedback after each trial. In the visual-exposure condition random feedback was given after each trial, hindering category learning while keeping visual exposure to the birds equal to the category-training condition. (B) In the pre and post-training fMRI scanning sessions the bird types were presented in blocks of five exemplars at morph ratios of 60∶40, 75∶25, and 90∶10. Each image was presented for 3 seconds with a mean inter-stimulus-interval of 1 s. Experimental blocks alternated with rest periods of 10 s. Subjects were instructed to view the birds attentively.

We designed a stimulus set consisting of six highly similar bird shapes that are difficult to distinguish without training (**Figure 1**). To directly test for neural correlates of category formation, a discrete category-boundary between similar-looking birds was established by training (**Figure 2a**). In addition to this categorization training, subjects performed a control task in which they were visually exposed to two other bird types, but to hinder category learning, the feedback they received was random [17]. Subjects were not informed that the feedback could be correct or incorrect. This manipulation allowed us to investigate neural changes specifically related to the formation of an object category compared with changes occurring as a result of repeated visual exposure. To investigate neural correlates of object-category formation, pre- and post-training fMRI time-series were obtained while the participants viewed exemplars of the different bird types (**Figure 2b**). We predicted that if category formation is mediated by increased neuronal responsiveness in occipitotemporal cortex, this increase should occur only for those birds for which a discrete category-boundary has been established, compared with visually similar birds for which no such boundary has been learned. Critically, this effect should be distinct from general training effects, such as increased familiarity and visual object-learning.

## Methods

### Subjects

Twelve neurologically healthy right-handed participants, not bird experts (ten females, mean age 20.7 years, range 18–25) with no neurological history participated in the experiment. All subjects had normal or corrected-to-normal vision. Subjects were paid for their participation. All subjects gave written informed consent. The study was approved by the local ethics committee (CMO region Arnhem-Nijmegen, the Netherlands).

### Stimuli

The stimuli consisted of pictures of birds that were constructed in a 3D model manipulation program (Poser 4 by Curious Labs, Santa Cruz, CA). First, six prototype birds were constructed from a base-bird (Songbird Remix by Daz3d, Draper, UT). Parts of the bird that were manipulated included its back, belly, tail, beak, head shape, cheeks, brow, and eye position. Next, each of the six birds was morphed with all other birds (at ratios of 95∶5, 90∶10, 80∶20, 75∶25, 70∶30, 65∶35, 60∶40, and 55∶45) analogous to the procedure used by Freedman and colleagues to investigate category formation in the monkey brain [18]. The category boundary was set at 50%. As a result, stimuli that were near opposite sides of a category boundary, though visually similar, belonged to different categories. Morphing happened smoothly between corresponding points on the birds. Each bird was colourless, rendered under the same lighting and camera settings, and exported as an image. Images had identical colour, shading and scale. In addition, using the same software, a set of control images of six different faces was constructed. The images measured 300 by 300 pixels in the training sessions and were slightly reduced in size (250 by 250 pixels) in the scanning sessions.

### Procedure and experimental paradigm

The six bird types were divided into pairs, and each pair was assigned to one of three conditions: 1) category training, where subjects received correct feedback to their responses, 2) visual exposure, where the amount of exposure to the birds was equal to the amount of exposure to the category trained birds, but category learning was hindered by random feedback, 3) no training. Assignment of bird types to the three conditions was counterbalanced over subjects in such a way that each bird type appeared equally often in each of the training conditions. The experiment was constructed using dedicated experimental software (Presentation by Neurobehavioral Systems, Albany, CA) and was run on a Pentium 4 with a 2.80 GHz processor and 2 GB of RAM.

#### Training

Training included three sessions, each of which lasted approximately two hours, on three consecutive days. During a training session, subjects sat comfortably in a soundproof cabin in front of a 19” computer screen. They performed a 1-back task on a series of bird images, in which they indicated with the index and middle finger of their right hand whether two consecutive birds were the same bird type or not. Subjects received feedback to their responses consisting of a printed text centred on the screen in coloured Arial font in size 16 (green: “right”, red: “wrong”, and yellow: “too late”). Bird exemplars were morphed at 55, 65, 70, 80, and 95% with all other bird types (e.g. bird type A at 95% morphed with B, C, D, E, and F at 5%). In total there were 25 exemplars (each bird type was morphed at five morph levels with the other five bird types) for each of the four bird types presented during training. Each exemplar was presented 30 times per training session. The average morph distance between birds was 58,67%. The proportion of same and different responses was fifty-fifty. In each trial, stimuli were presented for 1000 ms after which a response could be given during 2250 ms. Feedback was presented for 250 ms. Stimuli onset asynchrony was 4000 ms. A training session consisted of 10 blocks of 150 trials. In each block, 30 trials of category training (correct feedback) were alternated with 30 trials of visual exposure (random feedback). Subjects were not informed on this alternation of correct and random feedback conditions. Each block of 150 trials was followed by a small self-paced pause after which a subject could continue the experiment by pressing a button.

#### fMRI scanning

Subjects participated in an fMRI scanning session one day prior to training, and in an identical fMRI scanning session one day after training. During scanning, bird exemplars from each of the three conditions (category-training, visual exposure, and no training) were presented and subjects were instructed to view the birds attentively.

Bird exemplars were different from the exemplars encountered during training and included morphs at 60, 75, and 90%. Birds were presented in blocks. Each block contained 5 images of one bird type at a certain morph level. Images within one block were morphed with different bird types so that they were not identical to each other. For example, a block could consist of five images of 60% of bird-type A morphed with 40% of bird type B, C, D, E, or F. Each image was presented for 3 seconds with a mean inter-stimulus-interval of 1 s (varying between 600 and 1400 ms in steps of 200 ms between). Experimental blocks alternated with rest periods of 10 s for sampling the baseline. Experimental blocks were repeated six times, resulting in 108 blocks (6 bird types * 3 morph levels * 6 repetitions). In addition, six blocks were included that contained five images of artificial faces. Blocks were presented in pseudorandom order. Total scan time was 54.7 minutes.

Participants read the instructions for the scan session from a piece of paper before going into the scanner. They were instructed that they were going to watch pictures of objects presented in series of five and that these were followed by a few seconds of blank screen. They should watch these pictures carefully. To keep the subjects alert, we included catch trials. After each block a catch trial could occur. The chance of such an occurrence was on average, one out of six blocks. Subjects were instructed that once in a while, after the five pictures in the block were shown, an additional picture could appear after a cue. This picture was either an exemplar of the same bird type, but at a different morph level or an exemplar of a different bird type at the same or a different morph level as the bird exemplars in the previous block. They were instructed to judge whether this picture was the same bird type as the birds presented before the cue. The subjects indicated with a button-press on an MR-compatible response box (Lumitouch by Photon Control, Burnaby, Canada) whether this image was the same as the previously seen images (right index finger) or not (right middle finger). Subjects' heads were fixated and they were shielded from the scanner noise with earplugs. A beamer projected mirror-reversed stimuli on a screen at the end of the bore, which the subject was able to see through a mirror attached to the head coil.

### Imaging parameters

For each subject, 1575 whole-brain images (echo-planar imaging, 34 slices, 3 mm thick with 10% gap, repetition time = 2180 ms, voxel size = 3×3×3 mm, echo time = 30, flip angle = 70°, field of view = 19.2 cm, matrix size = 64×64) were acquired on a 3T whole body MR scanner (Magnetom TRIO by Siemens Medical Systems, Erlangen, Germany). In addition, a high resolution structural T1-weighted 3D magnetization prepared rapid acquisition gradient echo sequence image was obtained after the functional scan (192 slices, voxel size = 1×1×1 mm).

### Training data analysis

Response times for the correct trials and the percentage of correct trials were computed for each subject. These dependent variables were submitted to a training condition×morph level×session multivariate analysis of variance (MANOVA) with repeated measures. Training condition consisted of two levels (visual exposure and category training), morph level consisted of five levels (55, 65, 70, 80 and 95%), and session consisted of three levels (first, second, and third training session). To investigate the differentiation between training conditions over time, additional 2 (training condition)×5 (morph level) MANOVA's were performed for each of the training days. All significant interactions were explored with appropriate F-tests.

The presence of a category boundary was investigated by comparing the proportion of ‘same’ responses for bird pairs with an equal morph distance for cases in which the birds were from the same or from a different category. This was done for responses in the final training session, separately for the category training and visual exposure condition. Analyses of these data comprised a 2 (within or between category)×4 (10, 25, 30, 40 % distance) MANOVA for both the category training and visual exposure condition.

### fMR imaging data analysis

Imaging data analysis was done using BrainVoyager QX (by Brain Innovation, Maastricht, The Netherlands). The first two volumes were discarded to allow for T1 signal equilibrium. The following preprocessing steps were performed: slice scan time correction (using sinc interpolation), linear trend removal, temporal high-pass filtering to remove low-frequency non-linear drifts of 3 or fewer cycles per time course, and 3D motion correction to detect and correct for small head movements by spatial alignment of all volumes to the first volume by rigid body transformations. Estimated translation and rotation parameters were inspected and never exceeded 3 mm. Co-registration of functional and 3D structural measurements was computed by relating functional images to the structural scan, which yielded a 4D functional data set. Structural 3D and functional 4D data sets were transformed into Talairach space [19].

To dissociate between effects of training and effects of repetition we performed within-session analyses [12], [14]. Since objects were repeated in the training as well as in the control conditions, within-session differences between these conditions can not be due to repetition effects but must result from specific effects of training. Therefore, to examine specific training effects we compared responses to bird types in the different training and control conditions, separately for the pre- and post-training session.

Regressors of interest were modelled using a gamma function (tau of 2.5 s and a delta of 1.5) convolved with the blocks of experimental conditions [20] and multiple regression was performed using the general linear model (GLM). In order to correct for multiple comparisons, the false discovery rate (FDR) controlling procedure was applied on the resulting *p* values for all voxels. The value of *q* specifying the maximum FDR tolerated on average was set to .05. With this value, a single-voxel threshold is chosen by the FDR procedure which ensures that from all voxels shown as active, only 5% or less are false-positives [21], [22]. To further eliminate false-positives in the whole brain analysis, analyses were constrained to only those cortical areas that were responsive to viewing objects as compared with rest. To this end a conjunction analysis with a standard “minimal t-statistic” approach [23] was used, which is equivalent to a logical AND of the contrasts at the voxel level. For general training effects we used the contrasts: (Category training+Visual exposure<No training)∩(All objects>Rest) to detect training-related decreases in activity and (Category training+Visual exposure>No training)∩(All objects>Rest) to detect training-related increases in activity. For the specific effects of category training we used the contrast: (Category training>Visual exposure)∩(All objects>Rest) to detect increases in activity and (Category training<Visual exposure)∩(All objects>Rest) to detect decreased activity. To test for a main effect of session we contrasted (All objects pre-training)>(All objects post-training). All contrasts were calculated on data that were normalized using a z-transformation.

To further investigate responses within voxel populations (>50 mm^3^) that showed a significant effect of training, voxel-averaged beta-weights (i.e. regression coefficients) were extracted from these populations for each condition and morph level, separately for the pre- and post-training sessions and averaged over subjects. Random effects GLMs were computed using these regionally-averaged beta-weights. Specific effects of interest were tested with linear contrasts. All reported t-tests are two-tailed. The ROI time-courses were standardized, so that beta weights reflected the BOLD response amplitude of one condition relative to the variability of the signal.

To test for modulation of morph level we extracted the event-related responses to all bird conditions (category training, no training, and visual exposure) at all morph levels (10, 25, 40, 60, 75, and 90 %) from the region in the right middle fusiform gyrus that showed a category training effect. As an example, for the 10 % morph levels of category trained birds (if a subject had bird types A and B assigned to category training) we used responses to the following birds in the calculation: 90A:*10B*, 90C:*10B*, 90D:*10B*, 90E:*10B*, 90F:*10B*, 90B:*10A*, 90C:*10A*, 90D:*10A*, 90E:*10A*, 90F:*10A*. Each of these bird exemplars occurred six times in the experiment. In total there were 60 trials per morph level per condition. We then used ANOVA's to compute the linear relation between the morph levels and the brain response (beta weights).

## Results

### Training results

**Figure 3:**
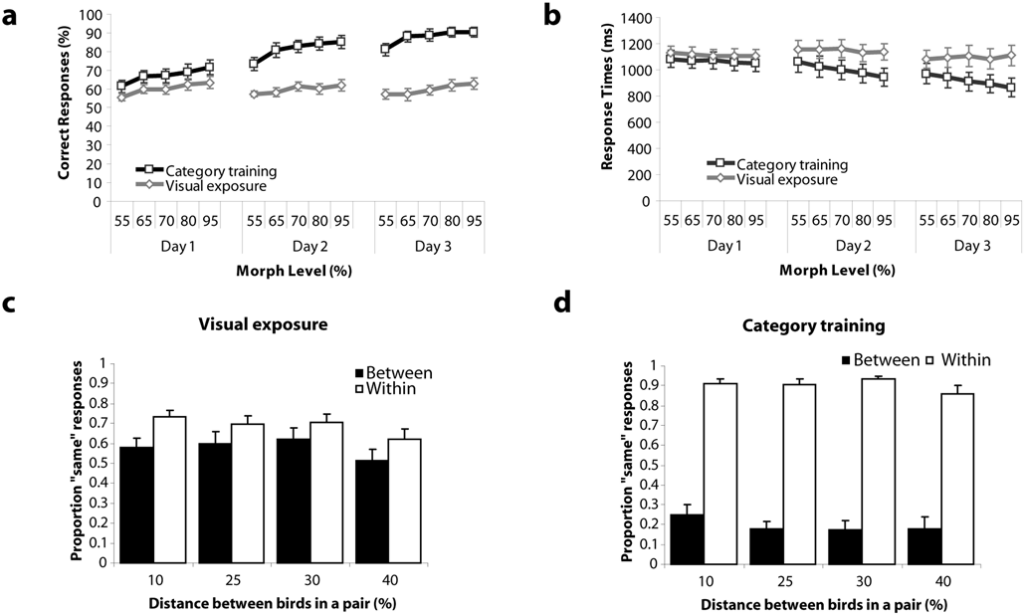
Training results. (A) Mean percentage of correct responses and (B) mean response latencies to the 1-back task, as a function of morph level, plotted for each of the three training days. (C, D) Proportion of “same” responses (see methods) as a function of physical distance between birds in a pair, separately for bird pairs that belonged to the same category (within) and bird pairs that belonged to different categories (between). The left histogram (C) presents the results for the category-training condition, the histogram on the right (D) the visual-exposure condition.

Behavioural training results showed that participants became proficient in categorizing the bird exemplars, but only after receiving correct feedback (**Figure 3, a and b**). In the first session, percentage of correct responses was equally low in both conditions [*F*(1,11) = 3.76, *p* = n.s.]. The percentage of correct responses increased as training progressed over time, but only in the category-training condition [*F*(2,10) = 29.27, *p*<.001, and not in the visual exposure condition [*F*(2,10) = 0.03, *p* = n.s.]. A similar pattern of results was found for response times. In the first session, no differences in response times were observed. Training-related decreases in response times were observed in the category-training condition [*F*(2,10) = 9.04, *p*<.01], whereas in the visual training condition response times remained stable over time [*F*(2,10) = 0.52, *p* = n.s.]. Significant differences in reaction times and accuracy between category-training and visual exposure conditions were obtained in session 2 (accuracy: [*F*(1,11) = 26.40, *p*<.001] reaction times: [*F*(1,11) = 8.60, *p*<.05]) and session 3 (accuracy: [*F*(1,11) = 40.45, *p*<.001]; reaction times: [*F*(1,11) = 5.80, *p*<.05]). By the end of training subjects had developed categorical perception for bird types trained with correct feedback. In the visual-exposure condition performance hovered between 55% and 65%. In the category-training condition, performance improved to around 90% correct for morphs close to the prototype. Even for morph ratios near the category boundary (55:45 morphs), performance exceeded 80% at the end of training. Thus, even though a 55:45 exemplar of, say, bird type A had only 55% of A properties (and 45% of either B, C, D, E, or F properties) it was nonetheless categorized as type A 80% of the time.

In the third training session, a significant effect of morph level [*F*(4,8) = 21.40, *p*<.001] was obtained. Responses were more accurate for bird exemplars with higher morph levels (close to the prototype) than for bird exemplars with lower morph levels (close to the category boundary). This effect of morph level was larger in the category training condition than in the visual exposure condition, as revealed by a condition×morph level interaction [*F*(4,8) = 6.02, *p*<.05]. In addition, responses were faster to bird exemplars closer to the prototype than to bird exemplars closer to the category boundary, but only in the category-training condition [*F*(4,8) = 6.87, *p*<.05].

The presence of a category boundary was investigated by comparing the proportion of ‘same’ responses for bird pairs with an equal morph distance for cases in which the birds were from the same or from a different category. This was done for responses in the final training session, separately for the category training and visual exposure condition. As expected, for category training we obtained a significant effect of the category boundary (**Figure 3, c and d**): Subjects were much more likely to rate bird pairs to be the same when they belonged to the same side of the category boundary than equal distance bird pairs belonging to different sides of the category boundary [*F*(1,11) = 115.86, *p*<.0001]. For visual exposure the effect was also present [*F*(1,11) = 4.97, *p*<.05] but smaller [*F*(1,11) = 5.22, *p*<.05]. Importantly, for category training there was no effect of physical distance [F(3,9) = 2.45, p<.05], and no interaction between distance and category boundary [*F*(3,9) = 0.88, *p* = n.s.]. The sharp difference in responses for within and between category pairs was maintained over decreasing physical distance between bird pairs (see **Figure 3c**), clearly indicating category formation. Furthermore, this result shows that the slightly greater performance for the more extreme morphs does not simply reflect a greater average distance between these morphs and their comparison stimuli. For the visual exposure condition a significant effect of distance [*F*(3,9) = 4.56, *p*<.05] was obtained. A higher proportion of ‘same’ responses was observed for bird pairs with a small distance than for bird pairs with a large distance (see **Figure 3d**). See **Text S1** and **Figure S1** for additional d prime analyses.

### fMRI results

Analyses of the pre-training fMRI data showed no significant differences in activity between the bird types. All birds elicited similar patterns of activity, indicating that initially, no differentiation between the birds was made on the basis of their physical features. To test for neural correlates of training-induced category formation, we analyzed post-training responses for the different bird types within object-responsive regions, that is, regions that were active for viewing objects as compared with rest (see methods). See also **Text S2** and **Figure S2** for the fMRI analysis and discussion of a main effect of session.

#### General effects of training

To test for general effects of training, we compared post-training fMRI responses to all trained bird types (category-training and visual-exposure conditions), with post-training fMRI responses to not-trained bird types.

**Figure 4:**
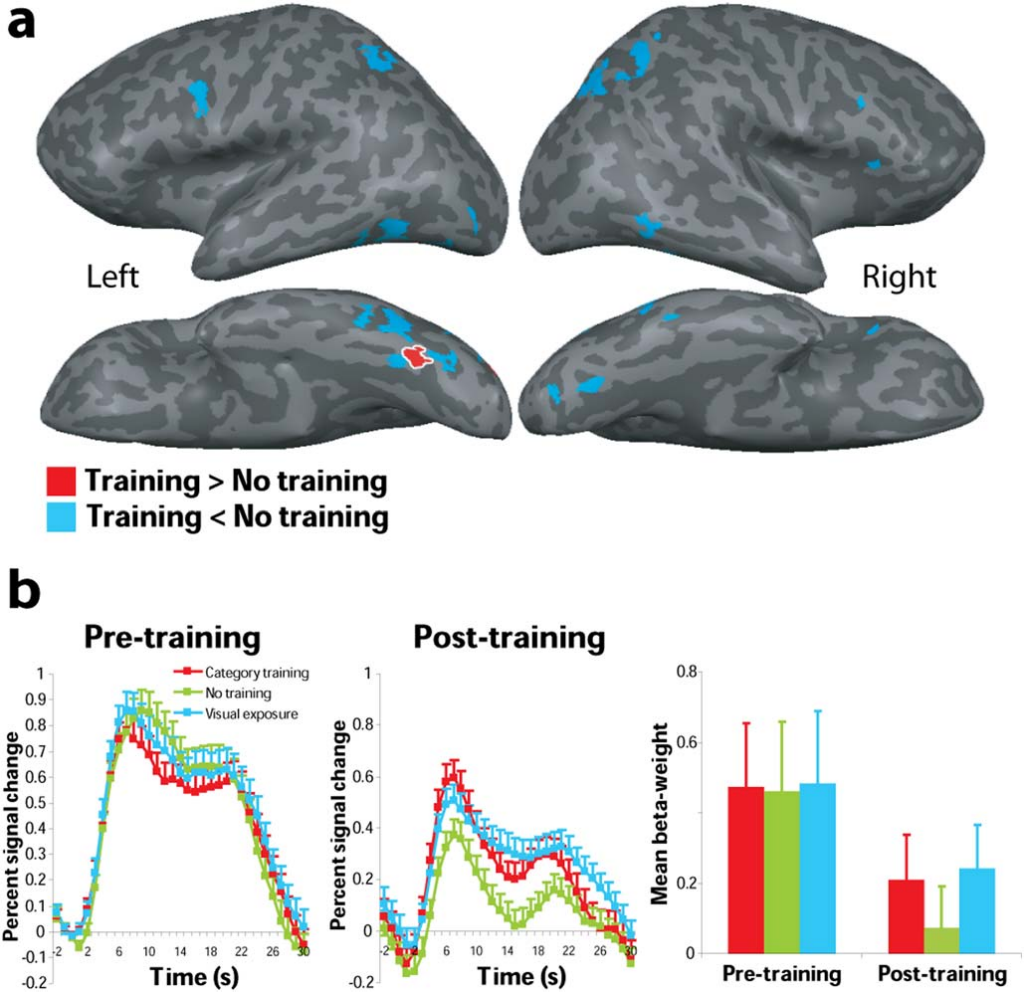
General effects of training. (A) Group-averaged activation maps from post-training scanning overlaid on lateral (top) and ventral (bottom) views of Talairach-normalized inflated hemispheres. In red, regions showing an effect of training as compared with no training at *p*<0.05 (False Discovery Rate corrected). In blue, brain regions showing decreased activity following training as compared with no training. (B) Group-averaged time-course of the BOLD response (percent signal changed) averaged over all voxels in the left fusiform gyrus (Talairach coordinates of the centre of mass: x = −33, y = −69, z = −18) that showed a general training effect. Shown are the group-averaged responses for each of three conditions in the pre and post-training scanning session (red: category training, green: no training, blue: visual exposure). Error bars represent standard error of the mean.

In the post-training session larger responses for trained compared with not trained bird types were obtained in the left posterior fusiform gyrus at a threshold of *p*<.05 (False Discovery Rate corrected) see **Figure 4a** and **Figure S3.** Additional random-effects multivariate analyses of the beta weights extracted from this region for each of the training conditions in both scanning sessions revealed a significant interaction between scanning session and training condition [*F*(2,10) = 10.64, *p*<.005]. The response to category-trained bird types was reduced in the post-training session compared to the pre-training session (t(11) = 2.90, p<.05, for the visual-exposure condition the response was also reduced but did not reach significance (t(11) = 2.00, p = .07). Whereas before training, conditions did not differ significantly, after training responses were significantly larger for training as compared with no-training conditions. Direct contrasts of post-training conditions showed that compared with no training, responses were enhanced in the category-training condition [*t*(11) = 2.58, *p*<.05] as well as in the visual-exposure condition [*t*(11) = 3.62, *p*<.005], see **Figure 4b**. In these voxel populations, no significant difference was found for category-training and visual exposure conditions [*t*(11) = 1.05, *p = *n.s.].

In addition to this general training-related enhancement of responses we observed general training-related decreases in activity in frontal, parietal, and occipitotemporal regions at a threshold of *p*<.05 (False Discovery Rate corrected), see **Figure S4** and **Table S1**. Additional random-effects analyses showed a significant interaction between scanning session and training condition in the right inferior temporal, bilateral fusiform, inferior occipital gyri, the right inferior and middle frontal gyrus, and the bilateral intraparietal sulcus. Whereas before training, conditions did not differ significantly, after training responses were significantly decreased for both for the category-training and the visual-exposure condition, as compared with the no-training condition (**Table S1**). In addition, these analyses revealed that these decreases in brain activity were independent of training condition. No differences were observed between responses in category-training and visual-exposure conditions.

#### Specific effects of category training

**Figure 5:**
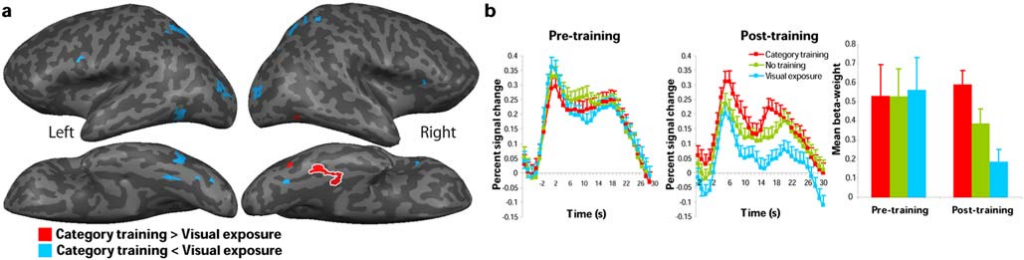
Specific effects of category training. (A) Group-averaged activation maps from post-training scanning overlaid on lateral (top) and ventral (bottom) views of Talairach-normalized inflated hemispheres. In red, regions showing a specific effect of category training as compared with visual exposure at *p*<0.05 (False Discovery Rate corrected). In blue, brain regions showing decreased activity following category training as compared with visual exposure. (B) Group-averaged time-course and mean beta-weights of the BOLD response in the right middle fusiform gyrus (Talairach coordinates of the centre of mass: x = 36, y = −35, z = −16) in percent signal change. Shown are the group-averaged responses for each of three conditions in the pre and post-training scanning session (red: category training, green: no training, blue: visual exposure). Error bars represent standard error of the mean.

To directly test for specific effects of category-training, we contrasted post-training responses to category-trained birds with post-training responses to visual-exposure birds. This contrast revealed significantly larger neural responses for category-trained birds in right middle fusiform gyrus and in the right lateral occipital gyrus (**Figure 5a**). A random effects analysis revealed significant greater activity for category-trained birds as compared with visual-exposure birds in the right fusiform gyrus [*t*(11) = 3.26, *p*<.01], but not in the lateral occipital gyrus [*t*(11) = 2.07, *p* = n.s.]. In addition to this increase in activity, decreases in activity for category-trained bird types as compared with visual exposure bird types were observed in occipitotemporal, inferior frontal, and parietal brain regions, see **Table S2 and** **Figure S5.** See also **Table S3** for areas that were more active for category training compared with no training and visual exposure compared with no training (and vice versa).

To further analyze the category-specific increase in activity, regions in the right middle fusiform gyrus showing a category-training related increase in activity were defined per subject (**Figure S6**). Mean beta-weights were extracted from these regions for each condition and morph level, separately for the pre- and post-training session (**Figure 5b**). A random-effects multivariate analysis of the regionally-averaged beta-weights showed a significant main effect of training condition [*F*(2,8) = 9.70, *p*<.01], as well as a significant interaction between session (pre- and post-training) and training condition [*F*(2,8) = 35.62, *p*<.0001]. Before training the right fusiform gyrus did not differentiate between the bird types. After training responses were significantly larger for the category-trained bird types than for visual-exposure and not-trained birds. Direct comparisons of the responses in the different training conditions revealed that responses for category-trained birds were significantly larger than responses for visual-exposure bird types [*t*(9) = 11.32, *p*<.0001], as well as not-trained bird types [*t*(9) = 3.06, *p*<.05]. In addition, significantly smaller responses were found for the visual-exposure condition as compared with the no-training condition (*t*(9) = 3.00, *p*<.05).

**Figure 6:**
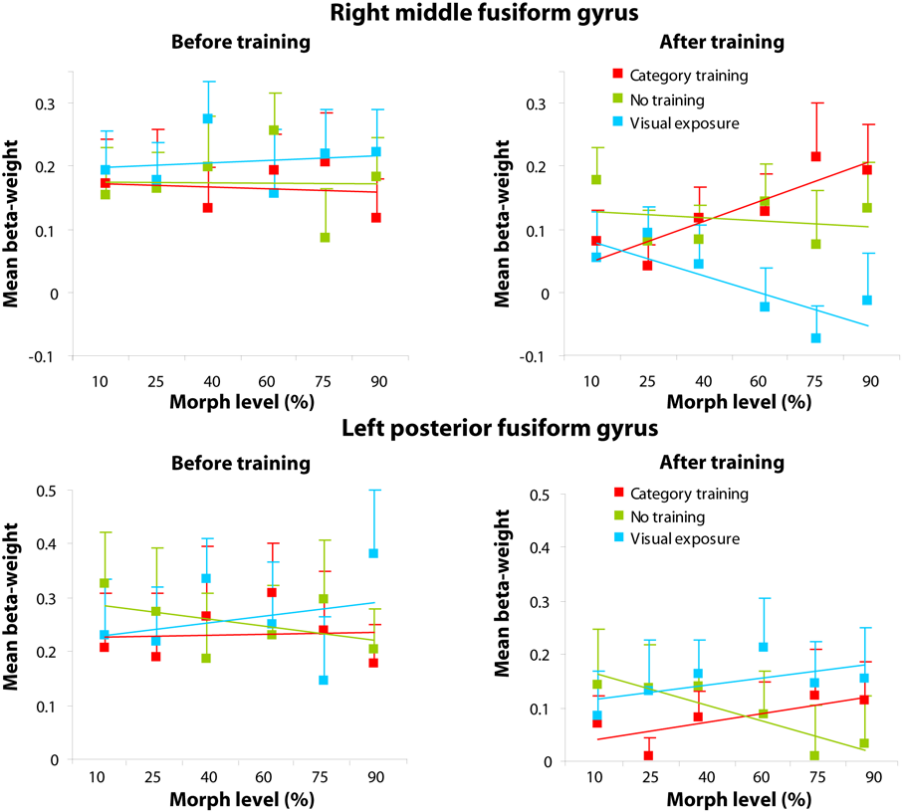
Fusiform responses as a function of morph level. The effect of morph level is plotted for voxels in the right middle fusiform gyrus showing a specific training effect and voxels in the left posterior fusiform gyrus that showed a general training effect in the post-training scan. For each training condition (red: category training, green: no training, blue: visual exposure) the regionally-averaged brain responses (mean beta-weight) are plotted as a function of morph level (%) in pre-and post-training scan sessions. Lines represent the optimal linear fit between morph level and brain response. Error bars represent standard error of the mean.

If the category-training related increase in the right middle fusiform gyrus is specifically related to sensitivity of neuronal populations to the diagnostic features of the category, we should see a positive linear relation between morph level and brain response. This relation should be present for the category trained birds, post-training but not pre-training, and also not for birds from the visual exposure condition for which category-learning was hindered. In addition, if the effect of morph level is specific to category learning it should not be present in the left fusiform gyrus, as this region showed a general training effect. To test this prediction, we investigated whether responses in the right middle and left posterior fusiform showed a linear increase as a function of morph level. As can be seen in **Figure 6**, a clear linear relationship of morph level and brain response was obtained in the post-training scan session for the category trained birds in the right fusiform only. Before training there was no linear relation between morph level and right middle fusiform response in the category training condition [*F*(1,4) = 0.09, p = n.s.; *R* = 0.15], birds from the no training condition [*F*(1,4) = 0.00, p = n.s.; *R* = 0.29], or for birds from the visual exposure condition [*F*(1,4) = 0.11, *p* = n.s.; *R* = 0.16]. After training there is still no linear relation between brain response and morph level for birds that were not trained [*F*(1,4) = 0.17, *p* = n.s.; *R* = 0.20]. However, for birds that were category trained there was a significant linear relation between morph level and beta-weight [*F*(1,4) = 15.87, *p*<0.05; *R* = 0.89] and interestingly for birds in the visual exposure condition there existed a negative linear relation between morph level and brain response [*F*(1,4) = 7.96, *p*<0.05; *R* = −0.82]. The responses in the left fusiform gyrus for category trained and visual exposure bird types showed no linear relation with morph level before [category training: *F*(1,4) = 0.11, *p* = n.s.; *R* = 0.05; visual exposure: *F*(1,4) = 0.30, *p* = n.s.; *R* = 0.27] or after training [category training: *F*(1,4) = 4.95, *p* = n.s.; *R* = 0.74; visual exposure: *F*(1,4) = 1.99, *p* = n.s.; *R* = 0.58]. This finding confirms that the effect of morph level in the right fusiform is specific for category learning and not a general consequence of training.

## Discussion

Our data provide evidence for experience-induced shaping of neural responses in ventral temporal cortex. Before training, all birds elicited similar patterns of activity, indicating that initially, no differentiation between the birds was made on the basis of their physical features. After training activity in occipitotemporal cortex was modulated as a function of experience. Post-training, activity in the left fusiform gyrus was significantly larger for trained as compared with not-trained bird types (**Figure 4**). This differentiation in responses occurred after category training as well as after visual exposure. Importantly, category training led to a relative increase in right fusiform responses. Post-training, bird types for which a sharp category-boundary was established during training elicited larger right fusiform responses than not-trained birds. In contrast, visual exposure alone resulted in reduced responses in the right fusiform gyrus (**Figure 5b**). This clearly shows that the increase in activity for category-trained bird types in the right fusiform gyrus was not caused by mere visual exposure, but mediates the formation of category-specific representations.

These results fit well with functional brain imaging data demonstrating increased activity in occipitotemporal cortex as a function of improved object recognition and visual expertise. Training-related increases in activity in occipital cortex have been reported to follow perceptual discrimination training with nonnatural nonsense objects [13], [14]. In addition, increased activity in the fusiform gyrus has been found after subjects became proficient in individuating a homogeneous set of nonsense objects [12]. Moreover, increased fusiform activity has been reported after subjects had learned to perform functional tasks with a set of novel stimuli [15]. In addition, larger fusiform responses were observed in individuals that were highly skilled in recognizing a particular class of objects such as birds, cars, or Lepidoptera (butterflies and moths) [24], [25], [26]. Although these results clearly show the involvement of occipitotemporal cortex in visual object learning they do not necessarily imply category formation. By dissociating between general effects of visual exposure and specific effects of category training we show that increased activity in the right fusiform gyrus is related to category formation.

Functional imaging data of humans [27] as well as electrophysiological recordings from monkey cortex [28], [29] have shown increased neural responses in ventral temporal cortex as a function of increased object familiarity. Recently, event-related potential data have shown distinct neural effects for object learning at basic and subordinate levels [30]. While training at a basic object level resulted in improved encoding of coarse visual features, training at a subordinate level resulted in additional encoding of more fine-grained visual object features. The present results show that on the first day of training, performance in the 1-back task was slightly above chance in both training conditions suggesting improved object coding as a function of visual experience. During the second and the third training session performance dramatically improved but only when subjects received correct feedback on their responses. This is in line with the idea that successful categorization of highly similar objects is mediated by learning fine-grained object features indicative of category membership. Indeed, whereas sensitivity in category discrimination was high for the category-trained bird types, for the visual-exposure bird types category-discrimination ability was very poor. In the visual exposure condition, the proportion of same responses was slightly higher for within- as compared with between-category bird-pairs. However, this small effect differed significantly from the sharp boundary effect obtained after category training. Consistent with the behavioural results, we found a clear neural dissociation between general effects of visual training and the formation of an object category. Whereas post-training training-related increases in activity in the left posterior fusiform gyrus occurred independently of category formation, increased responses in the right middle fusiform gyrus were only observed for bird-types for which a sharp category-boundary was established. This dissociation suggests that the left fusiform gyrus is probably involved in the encoding of general shape information, and the right fusiform is encoding fine-grained visual information required for category formation.

Our results are consistent with electrophysiological recordings from the inferior temporal cortex in monkeys suggesting that object category formation is mediated by a learning induced sharpening of neuronal stimulus selectivity [9], [10], [28]. Our behavioural data showed that responses were more accurate and faster for birds at higher morph levels, reflecting that birds close to the prototype are more distinctive than birds close to the category boundary. This implies that the closer to the prototype, the more apparent the features that determine to which category a bird belongs. Recently, it has been shown that neuronal selectivity in monkey inferior temporal cortex is shaped by those object features that were most relevant during categorization training [10]. In addition, single-cell recordings from monkey cortex have demonstrated that discrimination training enhances the selectivity of neurons in inferior temporal cortex not only for features in isolation but also for whole objects [11]. In line with these findings from monkey cortex, our findings suggest that after category training, neuronal populations in the right fusiform gyrus differentiated between object features that were informative of a category and features that were uninformative. Right fusiform activity was modulated by morph level (**Figure 6**). Responses were positively related with the morph-level of category trained birds and negatively related with the morph-level of birds for which category-learning was hindered by random feedback. This means that the higher the percentage of features trained to be relevant for categorization, the larger the responses in the right fusiform gyrus. In contrast, the higher the percentage of features trained to be irrelevant for categorization training, the smaller the right fusiform responses. Moreover, the left fusiform gyrus that showed a general training effect did not show a positive linear relation between morph level and responses, indicating that the effect of morph level is specific for category learning and does not occur as general consequence of visual exposure. One of the neural mechanisms that could explain this pattern of enhanced responsiveness to relevant category features and suppressed responses to irrelevant features involves increased tuning of neuronal populations to informative combinations of visual features. Op de Beeck et al. [14] have shown that the largest effects of training occur in regions that already process stimulus properties that are relevant during training. This suggests that increased tuning of neuronal populations concerns those features that were most relevant during training. However, since the present fMRI data reflect overall magnitude of response of relatively large neuronal clusters, no direct conclusions can be drawn on whether the results indeed reflect increased neural tuning. One way to investigate neuronal sensitivity with fMRI is by using an adaptation paradigm. Recent studies using this paradigm showed narrow shape tuning of neural populations in occipitotemporal cortex to sub-exemplar faces [31], [32] and trained car stimuli [16]. This suggests that neural populations in this brain region are highly specialized to dissociate between fine-grained visual features, which fits nicely with our interpretation of the results.

The location of our post-training training-related increase in activity in the right fusiform gyrus seems to be close to the location of the fusiform face area (FFA), a region that has been claimed to be specifically involved in face recognition [2], [33]. This claim has been challenged by findings relating FFA activity to increased expertise in object recognition [12], [24]. However, since we did not localize the FFA in our subjects we should be cautious about whether the current results directly address the debate regarding the function of the FFA. It is unclear whether the exact same region is involved here. The FFA is neighboured by regions that prefer other stimuli, such as bodies [34], [35]. Also, birds have faces and previous studies have shown that the FFA responds to animal faces to a considerable extent [36], [37]. Our subjects might have found the features in the bird's head extra useful for categorization. Therefore, the training-effects may have occurred in regions that process facial features. Note however, that not all features informative of a bird's category were located in its head and we cannot be certain that during training the facial features received indeed the most attention. Nevertheless, should the increase we observe for category-trained birds be attributed to the presence of a face in the stimuli, this does not deter from our novel finding of an increase that is specific to only those bird types for which category boundaries were formed during training.

In addition to training-related increases in activity, in some areas neural responses were significantly reduced for bird types from both category training and visual exposure conditions. These opposite patterns of responses in different brain regions might reflect two different learning mechanisms. While the underlying mechanism for the relative increase in the right middle temporal gyrus might be increased neuronal tuning for those object features relevant for category learning, a different mechanism could explain lower responses for trained compared with not-trained birds. Reduced occipitotemporal responses have consistently been reported to follow repeated exposure to visual objects [27], even over a delay of several days [38], [39]. This so-called repetition-suppression effect has been argued to reflect a learning process in which stimulus representations are optimized. Repeated exposure to the same stimulus causes neurons coding non-specific stimulus features to drop out of the responsive pool, while neurons tuned optimally to the stimulus continue their activity [40], [41], [42]. As a consequence, the total number of responsive neurons decreases, leading to a reduced overall response. In line with this idea, the reduced neural response for trained birds could reflect the formation of sharper object representations. Since reduced responses occurred in both the visual exposure and the category-training condition, this sharpening process is not related to object-category formation but probably reflects object-specific visual learning. In addition to general training-related decreases in activity, some occipitotemporal regions showed reduced responses for category-training as compared with visual-exposure conditions. This shows that applying random feedback not only hindered category learning [17], but also affected sharpening of object-specific representations. Although repetition suppression occurs as a result of repeated visual exposure, differences in encoding as a result of receiving correct or random feedback, might have led to differential changes in stimulus-specific representations [43], [44].

Our data provide evidence for learning-related formation of visual object category representation in occipitotemporal cortex. However, occipitotemporal cortex is not the only brain region that has been implicated in object-category learning. Monkey data have shown that neurons in prefrontal cortex respond selectively to members of a learned category, irrespective of within category variations [18]. These data were obtained while monkeys were actively involved in a categorization task. Although in our paradigm subjects may have been implicitly categorizing the birds throughout the scan session in order to successfully perform the task, this did not elicit training-specific increases in prefrontal cortex. Recently, it has been shown that prefrontal cortex shows a category-dependent response only when human subjects were performing a categorization task and not when performing a displacement detection task [16]. The exact relationship between the nature of a categorization task and category-selective responses in human cortex remains to be determined. Data from network models on object category learning suggest that during learning, the top-down influence of prefrontal cortex enhances the selectivity of the neurons in inferior temporal cortex encoding the behaviourally relevant features of the stimuli [45], [46]. Presumably, category-learning requires collaboration between these different brain structures, with the occipitotemporal cortex storing characteristic features of objects belonging to a learned category, and the prefrontal cortex being involved in explicit retrieval of category information.

## Supporting Information

Text S1 Training results: d' analysis.
(0.02 MB DOC)

Text S2 fMRI analysis of the main effect of session
(0.03 MB DOC)

Figure S1 **Training results.** Mean sensitivity (d') for the category-training and visual-exposure 1-back tasks as a function of the physical distance between two birds in a pair. Error bars represent standard error of the mean.
(0.54 MB TIF)

Figure S2 **Main effect of session.** Group-averaged activation maps of the between-session effect overlaid on lateral (top) and ventral (bottom) views of Talairach-normalized inflated hemispheres. In grey with a black outline, regions showing less activity for all objects after training as compared with activity to the same objects before training at *p*<0.01 (False Discovery Rate corrected).
(8.44 MB TIF)

Figure S3 **Single-subject data showing a general effect of training.** In red the areas that showed a higher response to trained as compared with not trained birds (*p*<0.05) overlaid on the axial slices from the corresponding normalized structural images. Structural images are in neurological convention.
(1.64 MB TIF)

Figure S4 **General effects of training.** **(A)** Group-averaged activation maps from post-training scanning overlaid on lateral (top) and ventral (bottom) views of Talairach-normalized inflated hemispheres. In red, regions showing an effect of training as compared with no training at *p*<0.05 (False Discovery Rate corrected). In blue, brain regions showing decreased activity following training as compared with no training. **(B)** Mean beta-weights (i.e., estimates of signal amplitude) for voxel populations in left and right occipitotemporal cortex showing a general decrease in activity for trained birds as compared with not-trained bird types. Shown are the group-averaged responses for each of three conditions in the pre- and post-training scanning sessions. Error bars represent standard error of the mean.
(4.49 MB TIF)

Figure S5 **Specific effects of category training.** **(A)** Group-averaged activation maps from post-training scanning overlaid on lateral (top) and ventral (bottom) views of Talairach-normalized inflated hemispheres. In red, regions showing a specific effect of category training as compared with visual exposure at *p*<0.05 (False Discovery Rate corrected). In blue, brain regions showing decreased activity following category training as compared with visual exposure. **(B)** Mean beta-weights for voxel populations in left and right occipitotemporal cortex showing a specific decrease for category-trained birds as compared with birds from the visual-exposure condition. Shown are the group-averaged responses for category-training, no training, and visual-exposure conditions in the pre- and post-training scanning sessions. Error bars represent standard error of the mean.
(3.65 MB TIF)

Figure S6 **Single-subject data showing a specific effect of category training.** In red the areas that showed a higher response to birds from the category training condition as compared with visual exposure birds (*p*<.05) in the right middle fusiform gyrus overlaid on the axial slices from the corresponding normalized structural images. Structural images are in neurological convention.
(1.30 MB TIF)

Table S1 Brain regions showing a significant decrease in activity after category training and visual exposure as compared with no training, as well as a significant interaction between training condition and scanning session in a random effects analysis. For each region, mean Talairach coordinates, corresponding Brodmann's areas (BA), averaged *t*-values (*df* = 11) for the contrast between (category training+visual exposure) and (no training) are reported, separately for the pre- and post-training sessions. In addition, averaged *t*-values (*df* = 11) are reported for the interaction between training condition and scanning session.
(0.05 MB DOC)

Table S2 Brain regions showing significantly less activity for category-trained birds as compared with birds from the visual exposure condition, as well as a significant interaction between training condition and scanning session in a random effects analysis. For each region, mean Talairach coordinates, corresponding Brodmann's areas (BA), averaged *t*-values (*df* = 11) for the contrast between category training and visual exposure are reported, separately for the pre- and post-training sessions. In addition, averaged *t*-values (*df* = 11) are reported for the interaction between training condition and scanning session.
(0.05 MB DOC)

Table S3 Table represents brain regions that showed significant (*p*<.05 FDR corr.) differences for contrasts that are not featured in the paper. For each region mean Talairach coordinates, volume in mm^3^, and averaged *t*-values for the relevant contrast are reported.
(0.10 MB DOC)
