# Supplementary material for: Birds of a Feather Flock Together: Experience-Driven Formation of Visual Object Categories in Human Ventral Temporal Cortex
Source: PLoS One. 2008 Dec 24;3(12):e3995. doi: 10.1371/journal.pone.0003995 (PMC2600611; doi:10.1371/journal.pone.0003995)
Supplement: Text S1 — Training results: d' analysis. (0.02 MB DOC) [file pone.0003995.s001.doc]

**Text S1**

**Training results**

Calculation of sensitivity (d’) in category discrimination showed that the average sensitivity was high for the category-trained bird types (d’ = 2.87) whereas for the visual-exposure bird types discrimination ability was very poor (d’ = 0.34), see **Figure S1.**
