# Supplementary material for: Birds of a Feather Flock Together: Experience-Driven Formation of Visual Object Categories in Human Ventral Temporal Cortex
Source: PLoS One. 2008 Dec 24;3(12):e3995. doi: 10.1371/journal.pone.0003995 (PMC2600611; doi:10.1371/journal.pone.0003995)
Supplement: Text S2 — fMRI analysis of the main effect of session (0.03 MB DOC) [file pone.0003995.s002.doc]

**Text S2**

**fMRI analysis of the main effect of session**

To test for a main effect of session we compared the responses to all objects post-training versus responses to all objects pre-training. The results showed that after training, responses to all objects were reduced compared with responses to the same objects before training in bilateral lateral occipital gyri extending into bilateral fusiform gyrus and inferior parietal cortex (see **Figure S2**). In addition we observed reduced responses in bilateral superior temporal sulcus, inferior and middle frontal gyri, and bilateral post and precentral gyri. Increased responses were observed in bilateral anterior and posterior cingulate gyrus and bilateral precuneus.

Because identical objects were used in the first and the second session, the overall differences in fMRI responses between the two sessions are likely to be related to repetition effects. It is by now well established that repeating an object (even over a delay of a few days) leads to decreases and increases in fMRI responses in a network of brain regions (e.g. [1-5]). The general effect of session that we observed is consistent with these findings.

1. van Turennout M, Ellmore T, Martin A (2000) Long-lasting cortical plasticity in the object naming system. Nat Neurosci 12: 1329-1334.

2. van Turennout M, Bielamowicz L, Martin A (2003) Modulation of neural activity during object naming: effects of time and practice. Cereb Cortex 13: 381-391.

3. Henson RN (2003) Neuroimaging studies of priming. Prog Neurobiol 70: 53-81.

4. Buckner RL, Goodman J, Burock M, Rotte M, Koutstaal W, et al. (1998) Functional-anatomic correlates of object priming in humans revealed by rapid presentation event-related fMRI. Neuron 20: 285-296.

5. Meister IG, Weidemann J, Foltys H, Brand H, Willmes K, et al. (2005) The neural correlate of very-long-term picture priming. Eur J Neurosci 21: 1101-1106.
