# Supplementary figures and images for: Birds of a Feather Flock Together: Experience-Driven Formation of Visual Object Categories in Human Ventral Temporal Cortex
Source: PLoS One. 2008 Dec 24;3(12):e3995. doi: 10.1371/journal.pone.0003995 (PMC2600611; doi:10.1371/journal.pone.0003995)

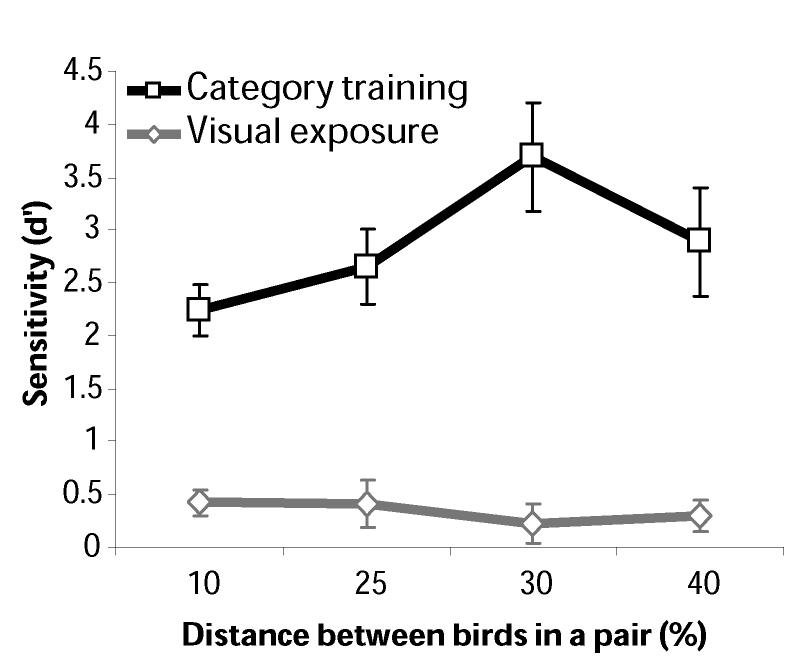

Supplement: Figure S1 — Training results. Mean sensitivity (d') for the category-training and visual-exposure 1-back tasks as a function of the physical distance between two birds in a pair. Error bars represent standard error of the mean. (0.54 MB TIF) [file pone.0003995.s003.tif]

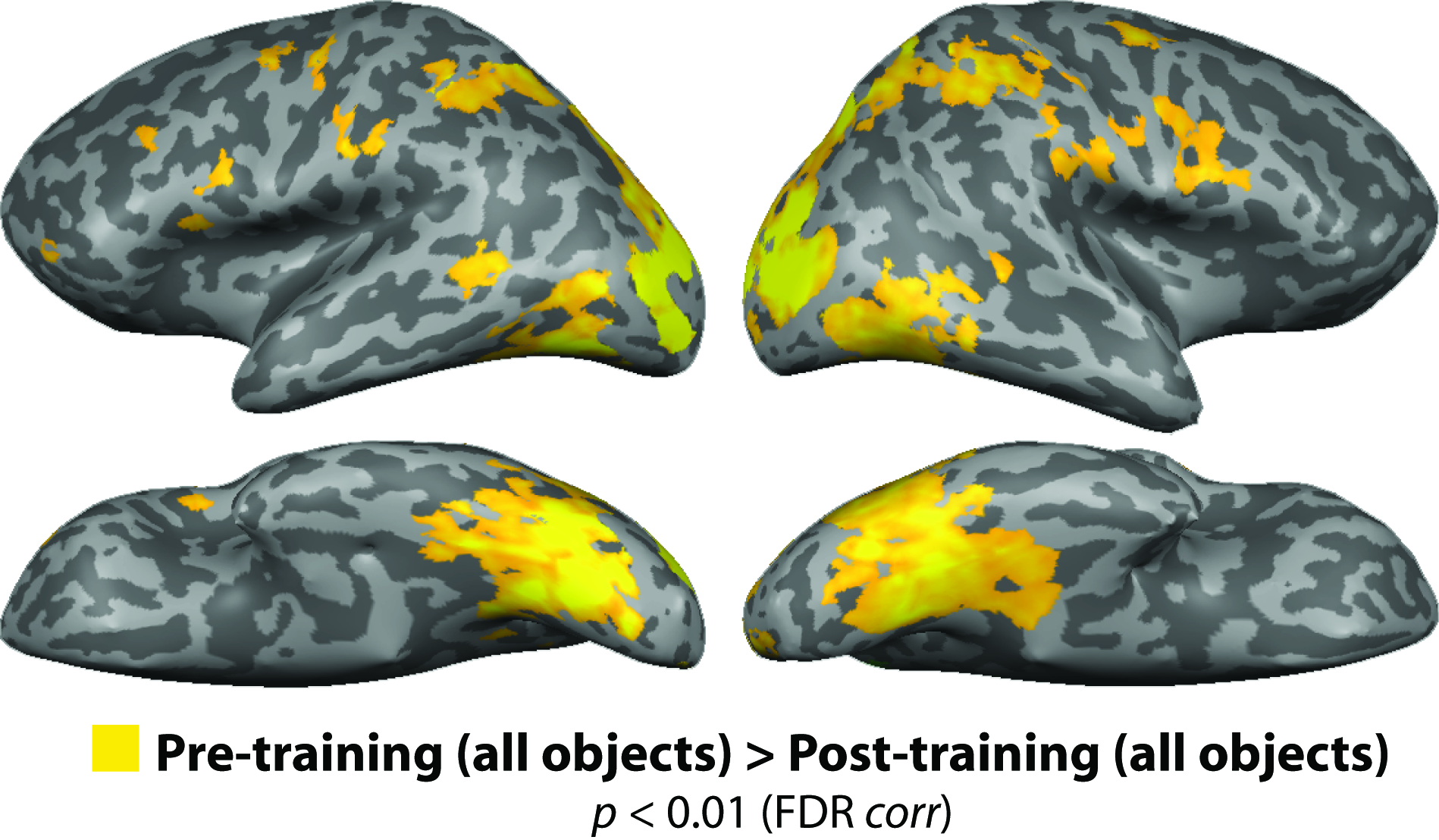

Supplement: Figure S2 — Main effect of session. Group-averaged activation maps of the between-session effect overlaid on lateral (top) and ventral (bottom) views of Talairach-normalized inflated hemispheres. In grey with a black outline, regions showing less activity for all objects after training as compared with activity to the same objects before training at p<0.01 (False Discovery Rate corrected). (8.44 MB TIF) [file pone.0003995.s004.tif]

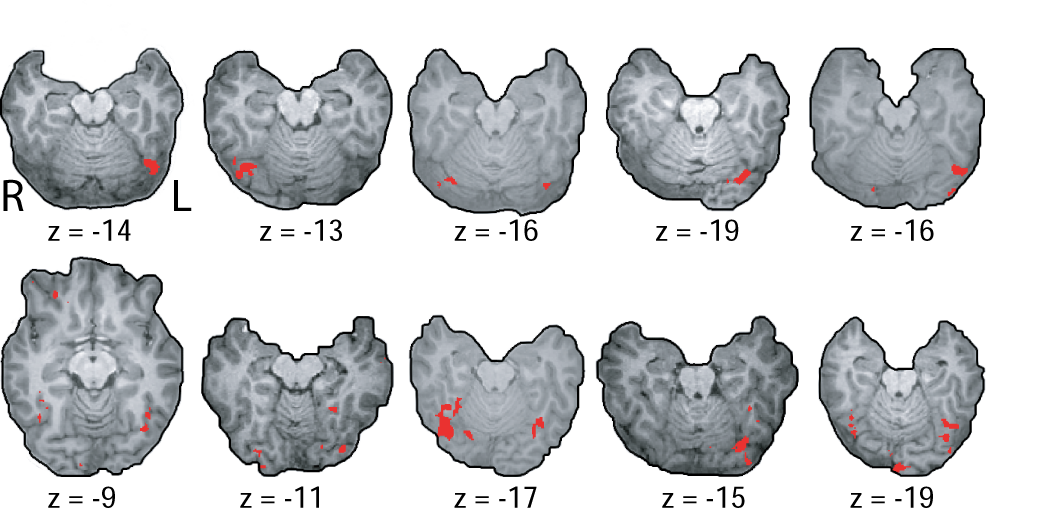

Supplement: Figure S3 — Single-subject data showing a general effect of training. In red the areas that showed a higher response to trained as compared with not trained birds (p<0.05) overlaid on the axial slices from the corresponding normalized structural images. Structural images are in neurological convention. (1.64 MB TIF) [file pone.0003995.s005.tif]

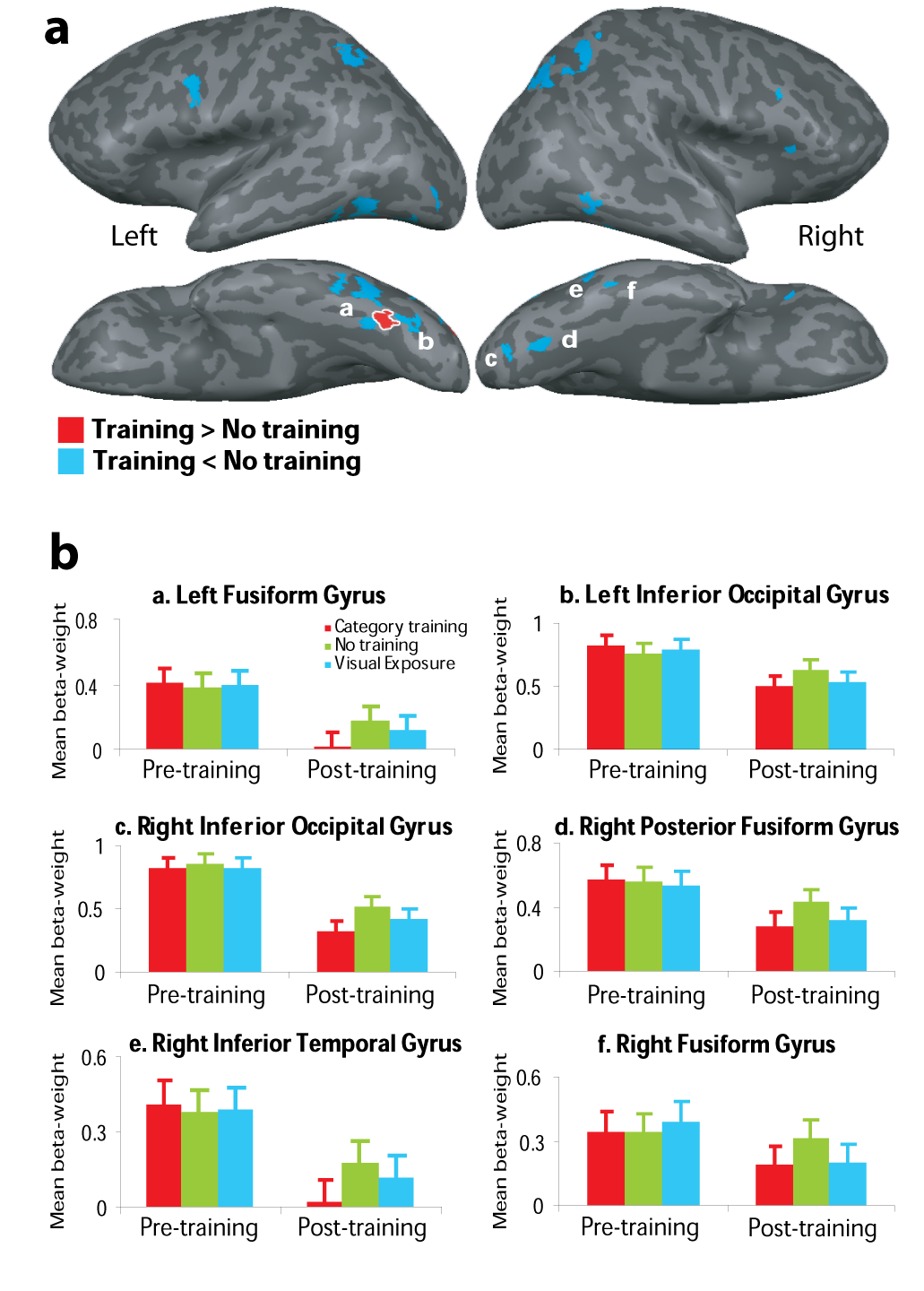

Supplement: Figure S4 — General effects of training. (A) Group-averaged activation maps from post-training scanning overlaid on lateral (top) and ventral (bottom) views of Talairach-normalized inflated hemispheres. In red, regions showing an effect of training as compared with no training at p<0.05 (False Discovery Rate corrected). In blue, brain regions showing decreased activity following training as compared with no training. (B) Mean beta-weights (i.e., estimates of signal amplitude) for voxel populations in left and right occipitotemporal cortex showing a general decrease in activity for trained birds as compared with not-trained bird types. Shown are the group-averaged responses for each of three conditions in the pre- and post-training scanning sessions. Error bars represent standard error of the mean. (4.49 MB TIF) [file pone.0003995.s006.tif]

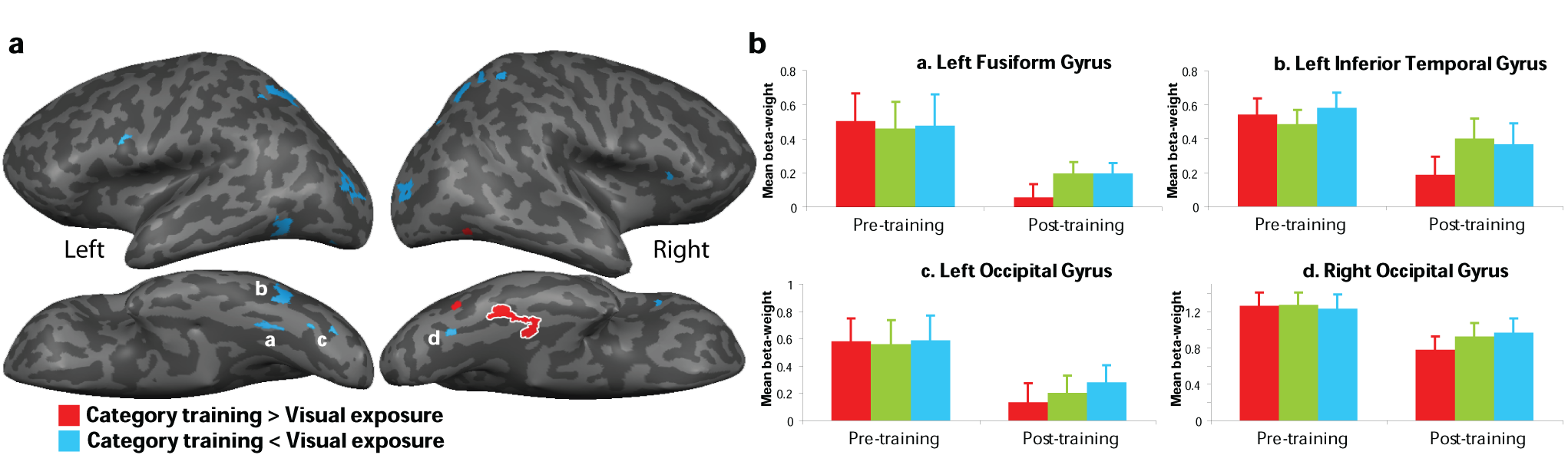

Supplement: Figure S5 — Specific effects of category training. (A) Group-averaged activation maps from post-training scanning overlaid on lateral (top) and ventral (bottom) views of Talairach-normalized inflated hemispheres. In red, regions showing a specific effect of category training as compared with visual exposure at p<0.05 (False Discovery Rate corrected). In blue, brain regions showing decreased activity following category training as compared with visual exposure. (B) Mean beta-weights for voxel populations in left and right occipitotemporal cortex showing a specific decrease for category-trained birds as compared with birds from the visual-exposure condition. Shown are the group-averaged responses for category-training, no training, and visual-exposure conditions in the pre- and post-training scanning sessions. Error bars represent standard error of the mean. (3.65 MB TIF) [file pone.0003995.s007.tif]

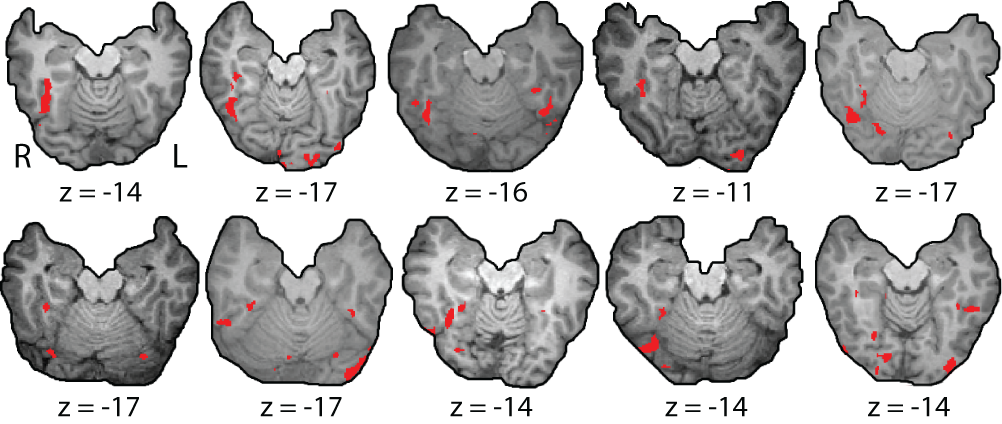

Supplement: Figure S6 — Single-subject data showing a specific effect of category training. In red the areas that showed a higher response to birds from the category training condition as compared with visual exposure birds (p<.05) in the right middle fusiform gyrus overlaid on the axial slices from the corresponding normalized structural images. Structural images are in neurological convention. (1.30 MB TIF) [file pone.0003995.s008.tif]
