# Supplementary material for: Birds of a Feather Flock Together: Experience-Driven Formation of Visual Object Categories in Human Ventral Temporal Cortex
Source: PLoS One. 2008 Dec 24;3(12):e3995. doi: 10.1371/journal.pone.0003995 (PMC2600611; doi:10.1371/journal.pone.0003995)
Supplement: Table S1 — Brain regions showing a significant decrease in activity after category training and visual exposure as compared with no training, as well as a significant interaction between training condition and scanning session in a random effects analysis. For each region, mean Talairach coordinates, corresponding Brodmann's areas (BA), averaged t-values (df = 11) for the contrast between (category training+visual exposure) and (no training) are reported, separately for the pre- and post-training sessions. In addition, averaged t-values (df = 11) are reported for the interaction between training condition and scanning session. (0.05 MB DOC) [file pone.0003995.s009.doc]

**Table** **S**1

| ROI | x | y | z | Pre-training | Post-training | Interaction  (session * condition) |
| --- | --- | --- | --- | --- | --- | --- |
| *Occipitotemporal* |  |  |  |  |  |  |
| Right Inferior Temporal Gyrus (BA 37) | 52 | -56 | -11 | 0.72^ns^ | 3.41** | 2.78* |
| Right Fusiform Gyrus (BA 37) | 48 | -46 | -14 | -0.68^ns^ | 4.08*** | 2.75* |
| Left Posterior Fusiform Gyrus (BA 37) | -34 | -60 | -14 | -0.83^ns^ | 3.88*** | 3.35** |
| Right Inferior Occipital Gyrus (BA 19) | 27 | -69 | -10 | 0.07^ns^ | 3.25** | 3.12** |
| Right Inferior Occipital Gyrus (BA 19) | 22 | -80 | 4 | 0.90^ns^ | 2.96* | 2.56* |
| Left Inferior Occipital Gyrus (BA 19) | -36 | -76 | -14 | -1.16^ns^ | 3.06** | 2.52* |
| *Frontal* |  |  |  |  |  |  |
| Right Inferior Frontal Gyrus (BA 45/46) | 41 | 29 | 17 | -0.65^ns^ | 3.43*** | 2.40* |
| Right Middle Frontal Gyrus (BA 9) | 46 | 15 | 35 | -1.35^ns^ | 2.19* | 3.05* |
| *Parietal* |  |  |  |  |  |  |
| Right Intraparietal Sulcus | 28 | -61 | 41 | -0.41^ns^ | 2.89* | 2.37* |
| Left Intraparietal Sulcus | -28 | -54 | 41 | -1.45^ns^ | 2.92* | 2.66* |

^ns^ not significant, * p < .05, **p < .01, *** p < .005
