# Supplementary material for: Birds of a Feather Flock Together: Experience-Driven Formation of Visual Object Categories in Human Ventral Temporal Cortex
Source: PLoS One. 2008 Dec 24;3(12):e3995. doi: 10.1371/journal.pone.0003995 (PMC2600611; doi:10.1371/journal.pone.0003995)
Supplement: Table S2 — Brain regions showing significantly less activity for category-trained birds as compared with birds from the visual exposure condition, as well as a significant interaction between training condition and scanning session in a random effects analysis. For each region, mean Talairach coordinates, corresponding Brodmann's areas (BA), averaged t-values (df = 11) for the contrast between category training and visual exposure are reported, separately for the pre- and post-training sessions. In addition, averaged t-values (df = 11) are reported for the interaction between training condition and scanning session. (0.05 MB DOC) [file pone.0003995.s010.doc]

**Table S2**

| ROI | x | y | z | Pre-training | Post-training | Interaction  (session * condition) |
| --- | --- | --- | --- | --- | --- | --- |
| *Occipitotemporal* |  |  |  |  |  |  |
| Left Inferior Occipital Gyrus (BA 19) | -21 | -82 | -20 | 0.58 ^ns^ | -3.89*** | -2.74* |
| Left Inferior Occipital Gyrus (BA 18) | -33 | -89 | 10 | 0.76 ^ns^ | -2.96* | -4.17*** |
| Left Fusiform Gyrus (BA 37) | -33 | -54 | -14 | 0.76 ^ns^ | -3.22** | -2.61* |
| Left Inferior Temporal Gyrus (BA 37) | -43 | -57 | -6 | -1.33 ^ns^ | -6.91*** | -3.92*** |
| Right Inferior Occipital Gyrus (BA 19) | 30 | -70 | -3 | 0.93 ^ns^ | -3.00* | -3.23** |
| Right Occipital gyrus (BA 18) | 18 | -94 | 6 | 0.67 ^ns^ | -2.62* | -2.64* |
| Right Inferior Occipital Gyrus (BA 18) | 27 | -83 | 7 | -0.41 ^ns^ | -2.63* | -2.28* |
| *Frontal* |  |  |  |  |  |  |
| Left Inferior Frontal Gyrus (BA 44) | -42 | 2 | 27 | 1.27 ^ns^ | -2.62* | -2.92* |
| Right Inferior Frontal Gyrus (BA 45) | 40 | 25 | 16 | -0.27 ^ns^ | 4.00*** | -3.28** |
| *Parietal* |  |  |  |  |  |  |
| Left Intraparietal Sulcus | -25 | -57 | 45 | 1.82 ^ns^ | -6.88*** | -6.13*** |

^ns^ not significant, * p < .05, **p < .01, *** p < .005
