# Supplementary material for: Birds of a Feather Flock Together: Experience-Driven Formation of Visual Object Categories in Human Ventral Temporal Cortex
Source: PLoS One. 2008 Dec 24;3(12):e3995. doi: 10.1371/journal.pone.0003995 (PMC2600611; doi:10.1371/journal.pone.0003995)
Supplement: Table S3 — Table represents brain regions that showed significant (p<.05 FDR corr.) differences for contrasts that are not featured in the paper. For each region mean Talairach coordinates, volume in mm3, and averaged t-values for the relevant contrast are reported. (0.10 MB DOC) [file pone.0003995.s011.doc]

**Table S3**

| ROI | | x | y | z | mm^3^ | t |
| --- | --- | --- | --- | --- | --- | --- |
| **Category training > No training** | |  |  |  |  |  |
| R occipital G | | 37 | -74 | -5 | 93 | 3.68 |
|  | | 6 | -74 | -15 | 89 | 3.72 |
| R fusiform G | | 39 | -59 | -9 | 71 | 3.78 |
| L fusiform G | | -30 | -70 | -18 | 159 | 3.61 |
| **No training > Category training** | | |  |  |  |  |
| R inferior temporal G | 53 | | -56 | -6 | 103 | 3.88 |
|  | 50 | | -57 | -16 | 162 | 3.47 |
|  | 47 | | -46 | -14 | 50 | 3.67 |
| R inferior frontal G | 50 | | 20 | 22 | 56 | 3.43 |
|  | 45 | | 16 | 34 | 157 | 3.51 |
|  | 42 | | 8 | 28 | 52 | 3.53 |
|  | 40 | | 28 | 17 | 1019 | 4.01 |
|  | 31 | | 42 | 13 | 54 | 3.32 |
| R middle frontal G | 42 | | 5 | 35 | 101 | 3.39 |
| R central S | 36 | | -34 | 47 | 102 | 3.20 |
| R insula | 36 | | -7 | 26 | 52 | 3.30 |
| R intraparietal S | 27 | | -60 | 43 | 3043 | 3.74 |
| R occipital G | 32 | | -86 | 0 | 204 | 3.34 |
|  | 26 | | -73 | -4 | 1388 | 3.63 |
|  | 21 | | -81 | -14 | 83 | 3.40 |
|  | 20 | | -94 | 5 | 113 | 3.65 |
|  | 12 | | -93 | 7 | 335 | 3.49 |
|  | 16 | | -76 | -8 | 53 | 3.37 |
| R fusiform G | 29 | | -50 | -13 | 55 | 3.40 |
| L occipital G | -17 | | -91 | -10 | 68 | 3.53 |
|  | -37 | | -76 | -14 | 212 | 3.76 |
|  | -34 | | -87 | 5 | 881 | 3.74 |
|  | -43 | | -78 | -2 | 98 | 3.42 |
| L intraparietal S | -27 | | -56 | 43 | 1560 | 3.60 |
| L inferior frontal G | -29 | | 47 | 9 | 54 | 3.28 |
|  | -36 | | 19 | 27 | 110 | 3.26 |
| L middle frontal G | -44 | | -1 | 32 | 863 | 3.62 |
| L fusiform G | -33 | | -55 | -14 | 257 | 3.74 |
| L inferior temporal G | -44 | | -56 | -8 | 1402 | 3.83 |
| L lateral occipital G | -49 | | -67 | 1 | 165 | 3.40 |
| **Visual exposure > No training** | | |  |  |  |  |
| L fusiform G | -35 | | -69 | -19 | 535 | 3.92 |
| L occipital G | -28 | | -92 | 11 | 58 | 3.58 |
| R occipital G | 32 | | -76 | -1 | 56 | 3.34 |
| **No training > Visual exposure** | | |  |  |  |  |
| R Inferior frontal G | 51 | | 22 | 23 | 88 | 3.82 |
|  | 39 | | 29 | 15 | 51 | 3.61 |
| L intraparietal S | -28 | | -53 | 36 | 123 | 3.72 |
| R inferior temporal G | 51 | | -57 | -14 | 70 | 3.70 |
| R occipital G | 11 | | -80 | -10 | 58 | 3.68 |
|  | 27 | | -70 | -12 | 57 | 3.70 |
|  | 36 | | -81 | -7 | 63 | 4.11 |
| R intraparietal S | 30 | | -54 | 43 | 284 | 3.81 |
|  | 28 | | -69 | 38 | 612 | 3.91 |
